# A case report of long-term successful stereotactic arrhythmia radioablation in a cardiac contractility modulation device carrier with giant left atrium, including a detailed dosimetric analysis

**DOI:** 10.3389/fcvm.2022.934686

**Published:** 2022-08-22

**Authors:** Mario Levis, Veronica Dusi, Massimo Magnano, Marzia Cerrato, Elena Gallio, Alessandro Depaoli, Federico Ferraris, Gaetano Maria De Ferrari, Umberto Ricardi, Matteo Anselmino

**Affiliations:** ^1^Department of Oncology, University of Turin, Turin, Italy; ^2^Division of Cardiology, Department of Medical Sciences, Città della Salute e della Scienza Hospital, University of Turin, Turin, Italy; ^3^Medical Physics Unit, Città della Salute e della Scienza Hospital, Turin, Italy; ^4^Department of Radiology, Città della Salute e della Scienza Hospital, Turin, Italy

**Keywords:** case report, stereotactic body radiation therapy, ventricular tachycardia, radiotherapy, cardiac contractility modulation

## Abstract

**Introduction:**

Catheter ablation (CA) is the current standard of care for patients suffering drug-refractory monomorphic ventricular tachycardias (MMVTs). Yet, despite significant technological improvements, recurrences remain common, leading to increased morbidity and mortality. Stereotactic arrhythmia radioablation (STAR) is increasingly being adopted to overcome the limitations of conventional CA, but its safety and efficacy are still under evaluation.

**Case presentation:**

We hereby present the case of a 73-year-old patient implanted with a mitral valve prosthesis, a cardiac resynchronization therapy-defibrillator, and a cardiac contractility modulation device, who was successfully treated with STAR for recurrent drug and CA-resistant MMVT in the setting of advanced heart failure and a giant left atrium. We report a 2-year follow-up and a detailed dosimetric analysis.

**Conclusion:**

Our case report supports the early as well as the long-term efficacy of 25 Gy single-session STAR. Despite the concomitant severe heart failure, with an overall heart minus planned target volume mean dosage below 5 Gy, no major detrimental cardiac side effects were detected. To the best of our knowledge, our dosimetric analysis is the most accurate reported so far in the setting of STAR, particularly for what concerns cardiac substructures and coronary arteries. A shared dosimetric planning among centers performing STAR will be crucial in the next future to fully disclose its safety profile.

## Introduction

Catheter ablation (CA) is the standard of care for patients suffering drug-refractory monomorphic ventricular tachycardias (MMVT) (1). However, despite significant technological advances, arrhythmic recurrences after CA remain common (2, 3). VT recurrences expose the patient to frequent readmission to intensive care units, psychological morbidity, progression of heart failure, and increased mortality (4).

New approaches have been proposed to improve the management of this highly challenging clinical condition, including neuromodulation and noninvasive VT treatment using radiotherapy (RT) (5–8). Stereotactic body radiation therapy (SBRT) applied to the heart, better known as STereotactic Arrhythmia Radioablation (STAR), is based on the precise delivery to a small volume of the heart of a single fraction of a high biologically effective dose of RT and has reported promising results (9, 10). STAR may overcome several limitations of conventional CA, which are strongly associated with VT recurrence, such as accessing regions of the heart chambers that cannot be reached with conventional CA (e.g., intramural scars or subepicardial locations). In addition, being noninvasive, STAR appears to be a safe alternative for most fragile patients (11).

As for conventionally fractionated RT, implantable cardioverter-defibrillator (ICD) or cardiac resynchronization therapy (CRT) carriers are eligible for STAR (12), although no firm conclusions can be currently drawn on the effects of thoracic stereotactic treatment on cardiac implantable electrical devices (CIEDs) patients, because of the lack of large prospective studies. A recent retrospective study (13) concluded that thoracic SBRT can be safely delivered when the dose to the CIED is kept below 2 Gy, the device is placed outside of the radiation beam, and the beam energy is ≤10 MV, irrespective of the pacing-dependency and of the CIED type (pacemaker or ICD). Therefore, by attending to these indications, CIEDs carriers can be eligible for STAR. No data, instead, are available on STAR in patients with cardiac contractility modulation (CCM), an emergent device for the management of patients with chronic heart failure and reduced ejection fraction (HFrEF) whose usage has become increasingly widespread in recent years (14). CCM aims at improving the strength of the cardiac contraction by generating relatively high-voltage (≈7.5 V), long duration (≈20 milliseconds), nonexcitatory biphasic electrical signals during the absolute myocardial refractory period. The system is constituted by one rechargeable implantable pulse generator and two active fixation leads secured to the right ventricular septum for sensing the ventricular activity and the bipolar delivery of the CCM pulses. The device has already been tested for potential interactions with ICD functioning (14).

We hereby present the case of a patient implanted with a CRT-D and a CCM device, treated with STAR for recurrent drug and transcatheter ablation resistant MMVT in the setting of advanced HFrEF and a giant left atrium, reporting 2-year follow-up and detailed dosimetric analysis.

## Clinical report

This case report was prepared following the CARE Guidelines (15); the Timeline is summarized in Supplementary Figure 1.

In April 2019, a 72-year-old Caucasian man was admitted at the Emergency Department with an ongoing MMVT (right bundle block with positive precordial concordance and inferior axis) at 185 beats per minute (bpm). The VT had started after a painful dental surgery including topic administration of adrenaline. Past medical history included permanent atrial fibrillation (AF) since 1968, mitral valvuloplasty due to rheumatic stenosis in the same year, followed by biological first (1986) and then mechanical (1995) valve prosthesis insertion; in 1998, a single lead pacemaker was implanted due to symptomatic slow ventricular response AF. The last cardiac ultrasound (US) performed in March 2019 showed left ventricle (LV) enlargement (188 ml, 66 mm) with a moderately depressed (40%) left ventricular ejection fraction (LVEF), giant left atrium (left atrial volume index, LAVI, of 989 ml/mq), regular mitral valve prosthesis functioning and no signs of pulmonary hypertension (PH). Outpatient functional class was relatively good (New York Heart Association, NYHA Class II) despite the concomitant presence of a severe restrictive respiratory disease requiring nocturnal bilevel positive airway pressure (BiPAP) therapy. The patient also suffered from chronic kidney disease (CKD). The VT was interrupted by electrical cardioversion (ECV) after intravenous amiodarone failure; LVEF in sinus rhythm was 30%, with a mild right ventricle (RV) dysfunction, a moderate tricuspid regurgitation (TR), and a mild PH. Coronary artery disease was excluded through angiography. The patient was discharged after pharmacological HFrEF therapy optimization.

**Figure 1:**
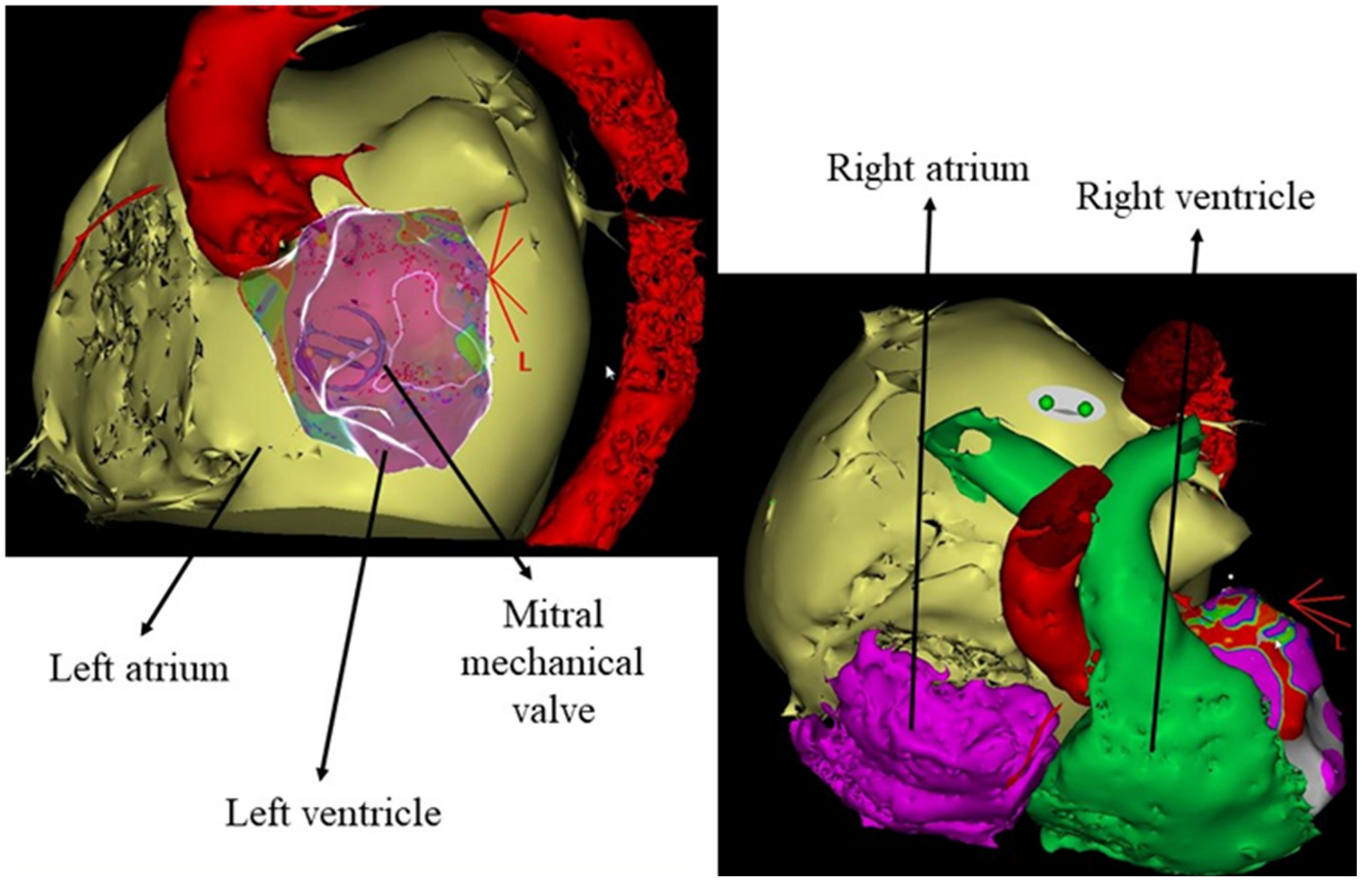
Anatomical details reconstructed from the preprocedural cardiac CT scan. Aorta in red, left atrium in yellow, right atrium in purple, and right ventricle in green.

One month later, the same MMVT relapsed at 165 bpm and was treated with ECV. Due to the concomitant respiratory disease, endocardial CA was preferred over chronic amiodarone treatment to prevent recurrences. Electroanatomic (EAM) activation and substrate maps were acquired (CARTO3, Biosense Webster, Irvine, CA, USA) and merged with pre-procedural CT scan (Figure 1). Late and fragmented potentials during sinus rhythm and mesodiastolic potentials during the clinical VT consistently pointed to a relatively restricted area located at a basal inferolateral region of the LV, which was targeted for ablation, leading to acute VT interruption and noninducibility at the end of the procedure. Due to the high percentage of RV pacing and the reduced LVEF, the patient subsequently underwent CRT-D implantation.

**Figure 2:**
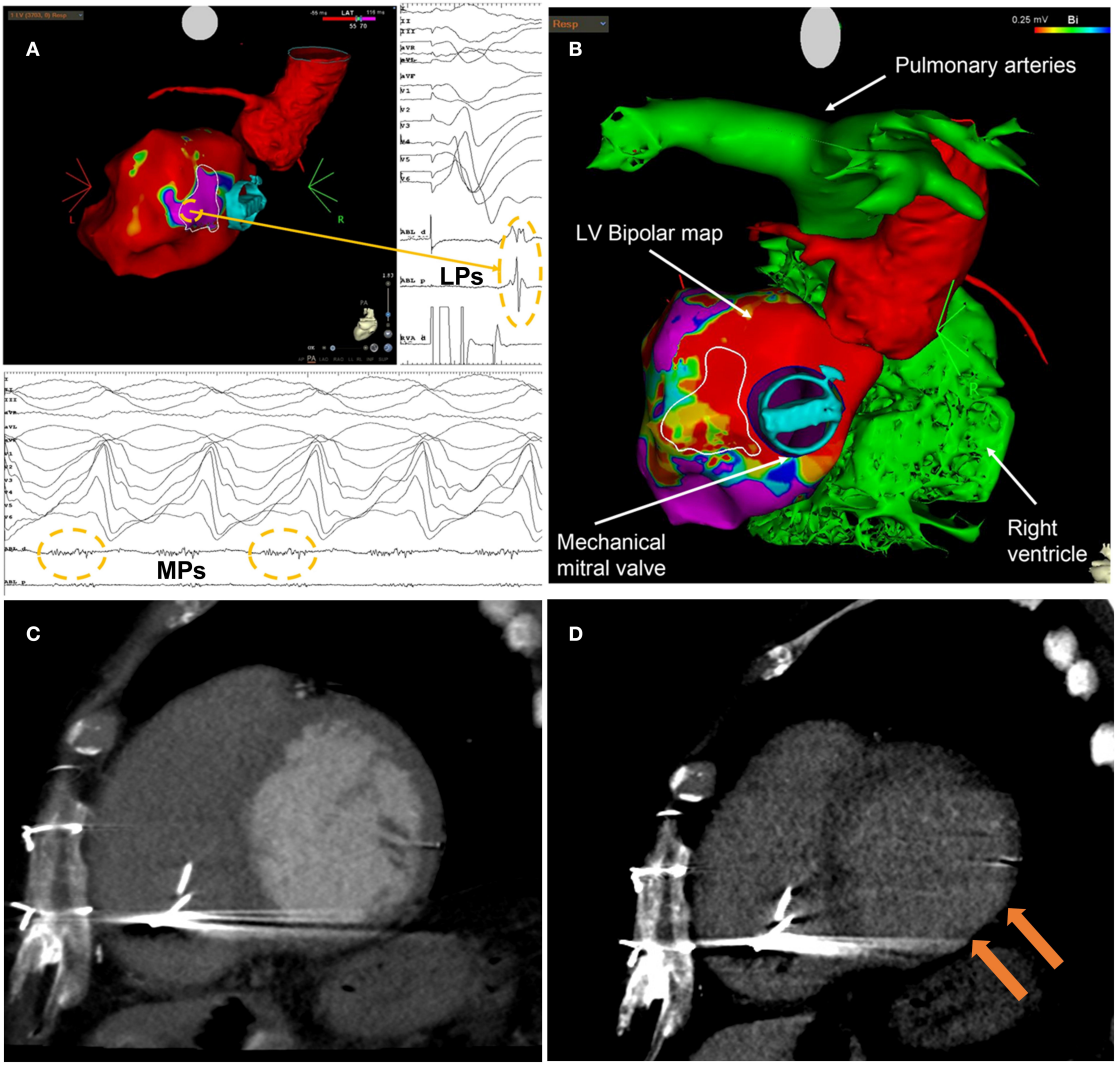
**(A)** Endocardial electroanatomic substrate map (CARTO3, Biosense Webster, Irvine, CA, USA) obtained from the second VT ablation procedure. The map highlights the area of late potentials (LPs) characterized by a local late activation time (LAT) after paced QRS end, located at the basal inferolateral segment of the left ventricle. Mesodiastolic potentials (MPs) recorded during the clinical VT were located at the same spot of the farthest LPs. **(B)** Integration of CT imaging with CARTO imaging data (bipolar voltage map). In both **(A,B)**, the white outlined area is the arrhythmogenic target for STAR identified at EAM mapping. **(C)** LV short axis view of cardiac CT angiographic phase with thinned basal inferolateral myocardium. **(D)** LV short axis view of late cardiac CT phase with hyperdensity on basal inferior-lateral wall that represents transmural fibrosis.

In July 2019, the clinical MMVT recurred, albeit slower (155 bpm) and below ICD detection, and was acutely interrupted during amiodarone infusion after several unsuccessful attempts of overdrive pacing. Despite the challenge related to a retrograde (through the aorta) only approach and navigation of the ablation catheter in the proximity of the mechanical mitral prosthesis annulus, a second endocardial CA procedure was carried out. Activation mapping during the clinical VT confirmed exit from the same spot identified at the first procedure (Figure 2A), which also showed an excellent 97% morphological matching during pacemapping in the site of mesodiastolic potentials; therefore, consolidation of the previous lesions at this spot was performed. Additionally, in an attempt to reduce the risk of recurrences, a subsequent substrate mapping during RV stimulation was performed, which led to the extension of the ablation lesions in the surrounding basal inferolateral area of the LV (white outlined area of Figures 2A,B) and, to a much lesser extent, to the anterolateral mediobasal regions of the LV (not shown in Figure 2). Again, non-inducibility was achieved at the end of the procedure. LVEF at discharge was 35% on nadolol 40 mg/die. In the following months, he was admitted to the hospital several times due to acute HF decompensation; cardiac US showed severe RV dysfunction and severe functional TR (Carpentier 1), not amenable to percutaneous correction. Therefore, in December 2019, he underwent uncomplicated CCM implantation, obtaining a subsequent transient functional improvement from outpatient NYHA class III to IIb.

Unfortunately, in February and March 2020, a total of seven episodes of the clinical MMVT recurred, both as isolated episodes and in form of electrical storms, with a mean heart rate of 140–145 bpm. Antitachycardia pacing (ATP) was not always successful, leading to ICD shocks. The patient was back to NYHA class III. Prophylactic amiodarone (200 mg/die) was started. Last cardiac US showed advanced biventricular dysfunction: LVEF 32%, RV fractional area change (FAC) 29%.

Due to the ineffectiveness of the two previous endocardial CA, the challenging anatomy (giant left atrium and mechanical mitral prosthesis), and the contraindication in approaching the epicardial side of the target area (previous cardiothoracic surgeries) without a new thoracotomy in a very fragile patient, he was referred for STAR. The patient provided informed consent for a compassionate-use protocol for STAR.

## STAR planning and procedure

An ECG-gated contrast-enhanced (CE) cardiac CT including myocardial delayed enhancement (DE) assessment and a CT scan in a supine position, using a dedicated device for immobilization (frameless Bluebag^®^ vacuum pillow), were obtained for planning purposes.

Cardiac CT was performed using a whole heart coverage (16 cm along with z-axis) CT scan (Revolution CT, GE Healthcare, Milwaukee, WI, USA) with the following parameters: slice configuration 256 × 0.625 mm, gantry rotation time 280 ms, and prospective electrocardiography (ECG) triggering. A new generation of iterative reconstruction was used for image reconstruction. The patient received a 1.5 ml/kg bolus of contrast medium (Visipaque 320 mg/ml, GE Healthcare), divided into two separate boluses of 5 ml/s followed by saline infusion. A first CT scan was obtained at the angiographic phase to have an adequate coronary artery opacification. A second series was acquired 7 min after contrast agent injection for the detection of myocardial DE. Visual evaluation of DE was performed using a narrow window width and level (350 W and 150 L) and a thick average intensity projection (0.5–0.8 cm). The presence of DE was confirmed as hyperdense myocardium with signal intensity >2 standard deviations above remote myocardium. A thinned basal inferolateral myocardium with transmural DE was identified (Figures 2C,D).

Free-breathing four-dimensional CT simulation (4D CT) allowed an assessment of the total cardiac and pulmonary motion. Ten CT phases distributed equally over the different phases of the breathing cycle were reconstructed for the 4D-CT data set. CE cardiac CT images were co-registered with those acquired during the simulation phase.

Definition of the gross tumor volume (GTV) was based on the combination of structural data from CE cardiac CT (wall thickness and DE) and EAM mapping data. Specifically, EAM mapping data from the two previous invasive endocardial CA procedures were combined to build a GTV for cardiac STAR that included the areas of previous ablations and the full myocardial thickness of the associated ventricular scar. Accordingly, the target volume was in the basal inferolateral region of the LV (white outlined area in Figures 2A,B). The contoured volume was strictly limited to regions of abnormal myocardium, either from a structural or an electrical point of view.

The GTV was defined using anatomical reference points such as the mitral valve and the interventricular septum. The LV was divided into basal, mid-cavity, and apex thirds by means of two plans parallel to the plane passing through the mitral valve. A further plan divided the basal third into two equal parts. Seven segments of the LV were identified: basal septal, basal lateral, mid septal, mid-lateral, apical septal, apical lateral, and apex. Additional plans perpendicular to the previous ones were placed to obtain a useful template with more reference points to guide the contouring of the target volume. The 3D reconstruction of the LV and of the contoured GTV, also including the ascending aorta and the prosthetic mitral valve, is shown in Supplementary Figure 2; the figure underlines the relationship between the GTV and the mitral valve. Once the GTV was contoured on a single series (CT 0%) of the 4D-CT, it was then moved to the other series and then adapted based on the LV displacement related to respiratory motion. All GTVs, contoured on the ten scans of the 4D-CT, were then moved to the average scan and summed altogether to generate an internal target volume (ITV) to compensate for the respiratory motion-related displacements of the target. An isotropic margin of 5 mm was added to the ITV to generate the planning target volume (PTV). The volume of GTV, ITV, and PTV were 26, 32, and 89 cc, respectively. With the aid of dedicated atlases (16–18), all organs at risk (OAR) including cardiac substructures were outlined on the average scan to estimate the average and the maximum cumulative radiation dose (Table 1). The enlarged left atrium with its 2,667 cc was contoured first. The co-registration between simulation CT and CE cardiac CT scans was used to contour all coronary arteries. A 3-to-5 mm expansion margin (PRV) was added to each coronary artery (CA-related PRVs) to cover the displacement due to cardiac motion and compensate for their motion, as previously reported (18, 19). Due to its proximity to the GTV, the mechanical mitral valve prosthesis was used as a landmark to identify the target volume and to measure the distance of the surrounding structures.

**Table 1:** Dosimetric parameters for organs at risk (OARs).

	**Dmax (Gy)**	**Average (Gy)**	**D_2%_**	**D_50%_**
Heart	29.21	3.15	24.00	1.35
Heart—PTV	19.77	2.69	15.53	1.30
Left ventricle	32.37	8.83	31.88	5.50
Right ventricle	5.98	1.56	5.57	0.68
Left atrium	16.78	2.41	13.05	1.25
Right atrium	2.92	1.06	2.70	1.01
Septum—Left ventricle	9.09	2.71	8.49	2.20
Free wall—Left ventricle	32.99	18.49	32.80	19.75
Aortic valve	0.83	0.47	0.79	0.44
Pulmonic valve	0.39	0.26	0.38	0.25
Mitral valve	27.69	13.23	27.10	11.90
Tricuspid valve	4.32	1.56	4.07	1.32
LMT	0.47	0.41	0.47	0.41
LAD	9.31	2.78	9.12	0.90
CFLX	32.50	18.67	32.43	29.34
RCA	1.28	0.34	1.16	0.28
Aorta arch	0.09	0.05	0.09	0.05
Aorta ascendent	0.36	0.16	0.34	0.15
Aorta descendent	2.96	0.62	2.82	0.22
Superior vena cava	0.23	0.19	0.22	0.18
Chest wall	13.71	2.60	12.87	0.67
Lungs	19.66	1.48	16.01	0.16
Left lung	23.54	2.40	19.43	0.24
Right lung	2.69	0.47	2.49	0.10
Trachea bronchus	0.37	0.08	0.35	0.03
Esophagus	5.86	1.49	5.65	0.48
ICD	0.04	0.02	0.03	0.02
CCM	0.01	0	0.01	0.00
Spinal cord	1.11	0.25	1.08	0.02

CCM, Cardiac contractility modulation; CFLX, Circumflex Coronary; D2%, dose received by 2% of the volume; D50%, dose received by 50% of the volume; Dmax, maximum RT dose; ICD, Implantable Cardioverter-Defibrillator; LAD, Left Anterior Descending Coronary; LMT, Left Main Trunk; PTV, planned target volume; RCA, Right Coronary Artery.

**Figure 3:**
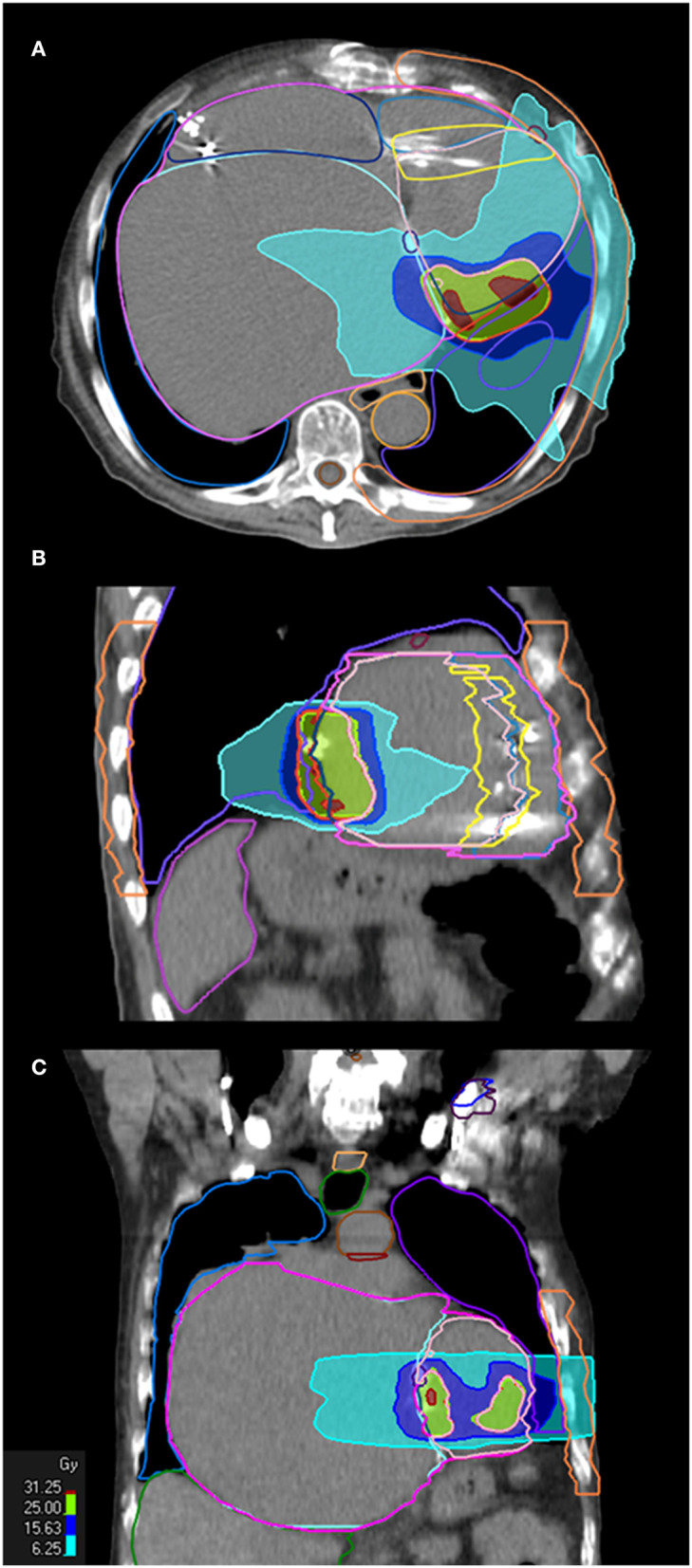
STAR treatment plan. Treatment plan in axial **(A)**, sagittal **(B)**, and coronal **(C)** orientation are shown, with dose volume histogram (25 Gy is prescribed on 80% isodose).

The prescription dose was 25 Gy in a single fraction. Ninety-five percent of the PTV was encompassed by the 80% prescription isodose. STAR was planned and then delivered with a volumetric modulated arc therapy (VMAT) solution. Ray Station software was used for treatment planning and the Monte Carlo algorithm for dose calculation. Two full arcs were delivered with flattening filter-free beams of 6 MV photons on an Elekta Versa Linear Accelerator (Elekta, Stockholm, Sweden). Figure 3 illustrates the dose distribution achieved with the VMAT plan. Notably, the average dose to the whole heart minus the PTV was well below the conventional 5 Gy safety threshold. Yet, due to the location of the target, the maximum and average dose to the circumflex artery were 32.5 and 18.67 Gy, respectively. The dose constraints of cardiac devices (12, 20) and all organs at risk (Table 1) were respected according to the latest recommendations for lung SBRT (21). Specific dosimetric constraints for CCM in patients undergoing SBRT are nonavailable yet; we thought reasonable using those recommended for pacemakers and ICDs.

Before treatment, image guidance using cone-beam CT (CBCT) was used to localize the target (22, 23). During RT treatment, audio-visual monitoring of the patient allowed intervention in case of necessity. Additionally, an emergency kit with an external defibrillator was available, and the treating cardiologists attended the treatment outside the linear accelerator (LINAC) room. Radiation delivery time was ~6 min. CRT-D and CCM devices were checked before and after irradiation.

## Clinical response and follow-up

STAR therapy was delivered in May 2020. The procedure was well-tolerated without sedation or anesthesia, and no acute complications occurred. The patient was discharged 3 days after, in stable conditions (NYHA class III).

After STAR, the patient was clinically evaluated at 1 month, and then every 3–4 months for the following 2 years. No more sustained, treated, or even not sustained ventricular arrhythmia episodes were documented with unchanged antitachycardia ICD programming as compared to before STAR. In September 2020, he was hospitalized for a few days due to acute decompensated HF and treated with diuretics, vasodilators, and levosimendan. At cardiac US, LVEF was stable (30%), but a mild further reduction in the already compromised RV function was noted (FAC 20 vs. 29%), accompanied by moderate PH (PAPs 40–45 mmHg compared to 20 mmHg). Nadolol was decreased from 40 to 20 mg/die and amiodarone from 1,400 to 1,200 mg/week. Also, CCM daily stimulation hours were increased from 9 to 14. A further amiodarone dose reduction has not been attempted yet due to the very fragile condition of the patient as well as the presence of polymorphic premature ventricular beats leading to a suboptimal (but stable on amiodarone) biventricular pacing percentage (94–95%) and potential CCM sub efficacy without amiodarone. No further HF decompensation episodes requiring hospitalization occurred thereafter. Cardiac US performed 23 months after STAR confirmed advanced but stable biventricular dysfunction (LVEF 28%, RV FAC 23%) with regular mitral valve prosthesis functioning, severe TR, and mild PH (PAPs 36 mmHg). No signs of leads or devices interference/damage/malfunction, as well as no clinical or radiological signs of late radiation-related complications, were observed (the patient underwent a chest CT scan in May 2022).

## Discussion

Our case report shows a complete and long-term VT suppression induced by STAR, applied on a compassionate use basis, in a very fragile patient with advanced HFrEF, giant left atrium, and a challenging VT substrate, implanted with a CRT-D and a CCM device, with no major safety concern. At the time we planned and performed the procedure, only a very small number of patients had been treated with STAR under experimental/compassionate protocols (24), with promising results but still several unsolved methodological and clinical issues. At present, only a minimum set of recommendations based on experts' opinion has been provided, which covers STAR indications and contraindications and monitoring during and after the procedure (25). Yet, optimizing the accuracy of each step from target definition to PTV generation, despite still far from being standardized, is critical to optimize treatment efficacy and safety. Accordingly, when looking at the largest case series of STAR published so far, most of the serious adverse events were clustered among the US-treated patients (9, 10, 26), who underwent STAR treatment on a significantly larger PTV [median PTV 98.9 cc, range, 60.9–298.8 for the 19 patients in the ENCORE-VT trial (10, 26)] than their European counterparts (27, 28) [mean PTV 34 ± 17 cc, range 12.8–62.1 cc, for the 18 patients described by Cvek et al. (28)].

Advanced arrhythmia source mapping for STAR has been performed based upon the results of invasive EAM mapping, electrocardiographic imaging (ECGi) with different systems (9, 26, 29–31), or even computational ECG mapping algorithms based on vectorcardiographic data analysis (32); to identify the GTV, electroanatomical data were then combined with anatomical and/or viability data obtained using different imaging techniques such as cardiac magnetic resonance imaging (9, 10, 33, 34), cardiac single-photon emission CT (9, 10), or CE cardiac CT (27, 30, 31, 33–35). Recent data suggest that enlarging the GTV to the entire potential arrhythmogenic substrates as identified by EAM substrate mapping may not provide further benefits compared to only targeting the critical isthmus of the clinical VT, while increasing side effects (36).

Concerning GTV contouring, manual transfer of the target volume to the RT treatment planning system by visual matching, as we did for our patient, is still the most used method. Yet, the use of a combination of manual transport and software-aided data review tools including semi-automated angulation and segmentation of the heart (37), or of in-house or open-source 3D data matching software (38) only, yield great potential for improvement.

Concerning the optimal compensation for cardiorespiratory movements of the thoracic targets and the reduction of the uncertainties related to patients' positioning, different methods have been proposed, including indirect cardiorespiratory tracking using fiducial markers such as the ICD lead (27, 28), optical surface monitoring system for continuous intrafraction positioning tracking (39), respiratory gating (29, 32) and even MRI-based cardiac gating (40). Considering the small number of patients treated with STAR and the heterogeneity of the local delivery platform and facilities, the benefit and the feasibility of each method are under evaluation. The last frontier in the real-time monitoring of cardiac motion during STAR is represented by an automatic cardiac US image acquisition system associated with an artificial intelligence algorithm (41).

The choice of the dose (25 Gy) was based on the preclinical and clinical data available at the time (24), suggesting a significant potential for myocardial fibrosis starting from 25 Gy and requiring at least 2–3 months to start to develop, with an acceptable safety profile. Notably, recent preclinical data (42) suggest an additional anti-arrhythmic mechanism for RT doses between 15 and 25 Gy, represented by electrical reprogramming leading to an increased conduction velocity, mostly due to an increased expression of NaV1.5 channels and the gap-junctional protein Cx43. This functional effect was observed in animals early after a single RT treatment, but there are no data concerning its long-term durability in the control of ventricular arrhythmias. Accordingly, in several cases (24, 29, 34, 35) including the present one, the anti-arrhythmic effects of STAR were observed immediately after the procedure, with no blanking period. Our dosimetric analysis is the most accurate reported in the setting of STAR, particularly for what concerns cardiac substructures and coronary arteries (18, 19). Notably, the 2010 Task Group report on dose constraints for SBRT treatments (43) does not contain any limitation for cardiac substructure, due to the lack of significant correlations to treatment-related side effects in the available literature. For some of these substructures (not including cardiac valves), the ongoing RAVENTA trial (44), a clinical trial for STAR treatments, suggests specific dose limits, in particular a maximum dosage to the left arteries of 20 Gy, that was not attended in our patient. All the other suggested constraints were respected. A detailed and shared dosimetric planning among centers performing cardiac SBRT will be crucial in the next future to fully disclose its safety profile.

In conclusion, our case report supports the early as well as long-term efficacy of 25 Gy single-session STAR. Despite the concomitant severe HFrEF, with an overall heart-PTV mean dosage below 5 Gy, no major detrimental cardiac effect within 2 years was registered. Yet, it must be acknowledged that basal, perivalvular targets irradiation may lead to late native valve toxicity or coronary damage. In addition, despite encouraging preliminary results of STAR, a significant number of treated patients all over the world were reported to suffer VT recurrences. Whether this is due to inaccurate VT substrate delineation (incorrect target), inaccurate transfer to the treatment planning system or inaccurate or insufficient radiation delivery remains to be elucidated. Translational research, prospective clinical trials, and International consortiums such as the ongoing STOPSTORM,^1^ founded by a Horizon 2020 grant, will be crucial in the next future to fully unravel the dose–response issue of cardiac SBRT and to standardize treatment planning and delivery as well as patient's selection and data collection, to fill the actual gaps in knowledge and optimize the efficacy and safety of the procedure.

## Data availability statement

The original contributions presented in the study are included in the article/Supplementary material, further inquiries can be directed to the corresponding author/s.

## Ethics statement

Written informed consent was obtained from the participant/s for the publication of this case report. Written informed consent was obtained from the individual(s) for the publication of any potentially identifiable images or data included in this article.

## Author contributions

ML, VD, MM, MC, EG, AD, FF, GD, UR, and MA contributed in different ways to patient's management, STAR planning and/or delivery. ML, VD, and MM wrote the first draft of this manuscript. All authors discussed the results, contributed to improved draft versions, and approved the final version of the manuscript.

## Conflict of interest

The authors declare that the research was conducted in the absence of any commercial or financial relationships that could be construed as a potential conflict of interest.

## Publisher's note

All claims expressed in this article are solely those of the authors and do not necessarily represent those of their affiliated organizations, or those of the publisher, the editors and the reviewers. Any product that may be evaluated in this article, or claim that may be made by its manufacturer, is not guaranteed or endorsed by the publisher.

## Footnotes

^1^ https://www.stopstorm.eu/
