# Supplementary material for: A case report of long-term successful stereotactic arrhythmia radioablation in a cardiac contractility modulation device carrier with giant left atrium, including a detailed dosimetric analysis
Source: Front Cardiovasc Med. 2022 Aug 22;9:934686. doi: 10.3389/fcvm.2022.934686 (PMC9441661; doi:10.3389/fcvm.2022.934686)

Supplemental figure 1: Timeline according to CARE indications.

Supplemental figure 1 abbreviations: AF=atrial fibrillation; bpm= beats per minute; CA=catheter ablation; CCM= Cardiac contractility modulation, CRT-D=cardiac resynchronization therapy defibrillator; NYHA= New York Heart Association Class; LAVI=left atrial volume index; LVEF= left ventricular ejection fraction; MMVT=monomorphic ventricular tachycardia.

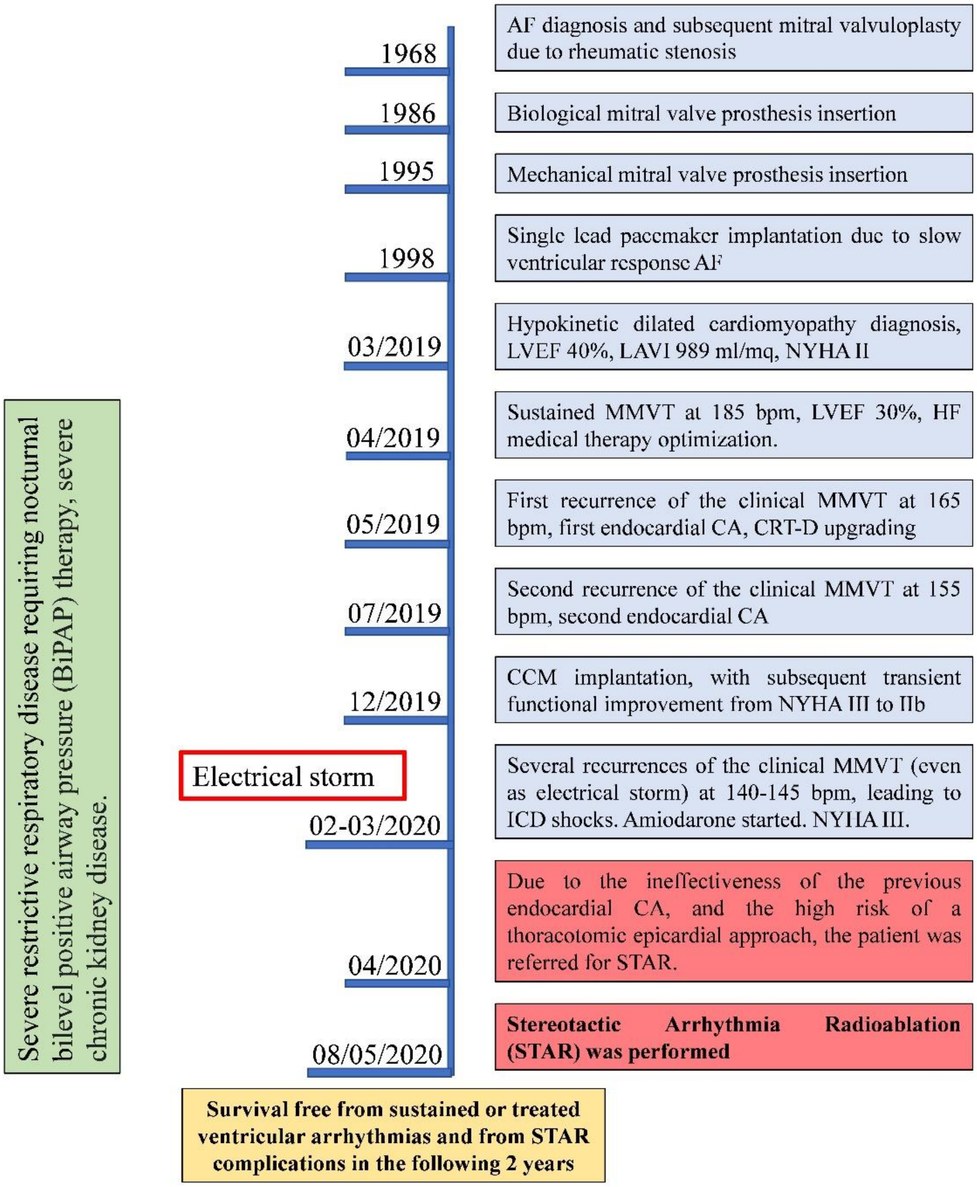

Supplemental figure 2: 3D reconstruction of the LV and of the contoured GTV. The figure underlines the relationship between the GTV and the mitral valve. GTV in pink, Left Ventricle in blue, Mitral Valve in light blue, Ascending Aorta in red.

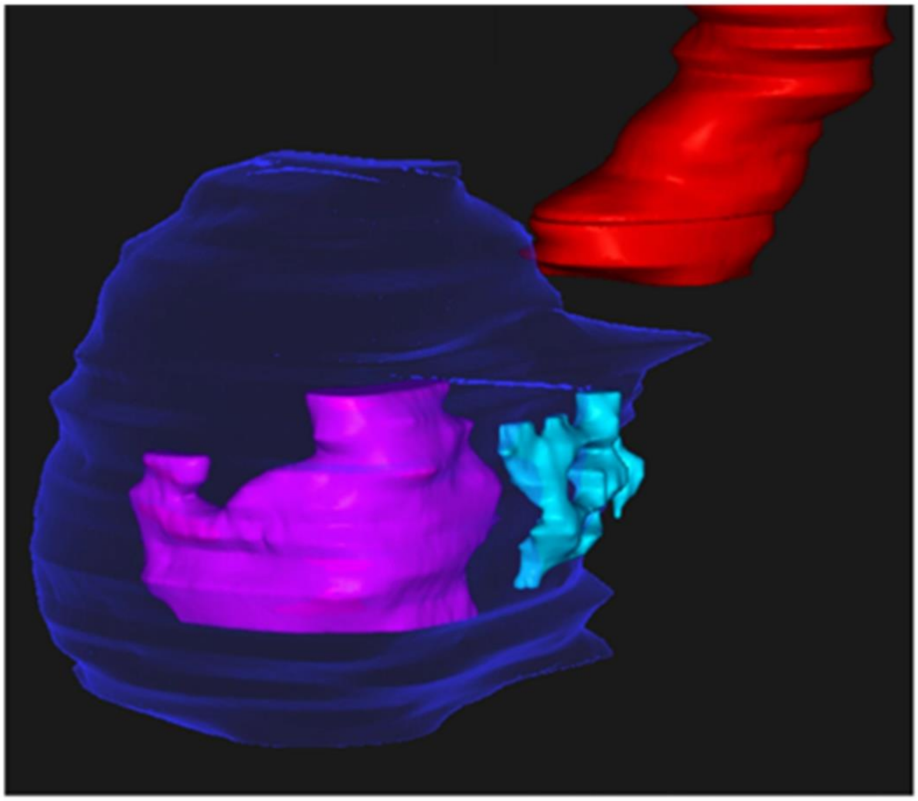

Supplement: Supplementary file 1 [file Presentation_1.pdf]
